# Be Aware of the Benefits of Drafting in Sports and Take Your Advantage: A Meta-Analysis

**DOI:** 10.1155/2023/3254847

**Published:** 2023-11-08

**Authors:** Floor A. P. van den Brandt, Mohammed Khudair, Florentina J. Hettinga, Marije T. Elferink-Gemser

**Affiliations:** ^1^Department of Human Movement Sciences, University Medical Center Groningen, University of Groningen, Groningen, Netherlands; ^2^Department of Sport, Exercise & Rehabilitation, Faculty of Health and Life Sciences, Northumbria University, Newcastle-upon-Tyne, UK

## Abstract

**Purpose:**

In competitive sports, optimizing performance is the key. An interesting venue to explore is to consider drafting as a pacing strategy. The purpose of this study is to identify the magnitude of drafting benefits for biomechanical, physiological, and psychobiological parameters in and between athletes in cycling, kayaking, running, skating, skiing, and swimming.

**Design:**

A systematic review and meta-analysis.

**Methods:**

Systematic searches were performed in PubMed, Web of Science, and Embase databases.

**Results:**

In total, 205 studies were found, from which 22 were relevant (including 232 participants and 548 observations). Methodological quality was high for all the included articles. The meta-analyses for all parameters indicated strong evidence for a benefit of drafting, with moderate effects between leading and drafting athletes found for the heart rate (3.9%), VO_2_ (8.9%), power output (11.3%), and rating of perceived exertion (10.4%). Large effect sizes were found for blood lactate (24.2%), VE (16.2%), and EMG (56.4%). A moderator analysis showed differences between sports on the effect of drafting with most benefits in cycling. *Discussion*. Based on the observed effects of drafting in the biomechanical, physiological, and psychobiological parameters, it can be considered as an element of pacing, a strategy to conserve energy and optimize performance.

**Conclusion:**

There is strong evidence that drafting benefits athletes, with varying levels of effect for athletes in different sports. Knowledge about the magnitude of benefits can be used to improve training sessions, race strategies, and performance in competition.

## 1. Introduction

Drafting is the phenomenon where a moving object follows closely behind another moving object to reduce the air and water resistance [1]. Air resistance or drag force is the force which acts in the opposite direction to the relative motion of any object with respect to a surrounding fluid [2]. Drafting is also called slipstreaming or moving in the sheltered position and is often used in nature, where, for example, birds migrate in V formation. It is also often used in sports when athletes move together in groups [1]. It is a multidimensional topic where biomechanical, physiological, and psychobiological parameters are important because they appear to all be decreased for the drafting athlete [3, 4]. Unknown, however, is the magnitude of this reduction for drafting athletes in a variety of sports. At the elite level, the difference between winning the gold medal and missing the medal ceremony can be very small. All information that can help improve performance is of utmost importance, especially since the stakes in the international sporting arena at the world-class level are high. Improving drafting skills could be one of the “hidden” performance characteristics that receive less attention in practice but holds the potential to increase performance. Therefore, the tactical use of drafting can be very interesting for athletes and coaches to optimize performance. The current review will give an overview of the available research on the effect of drafting and the differences between sports and discuss why, how, and when to use drafting to gain full advantage so that the benefits of drafting can optimize performance.

Drafting decreases the drag force on an athlete, which is the resistance force caused by the motion of an athlete through a fluid, such as water, or through air [5]. Friction drag occurs due to the interaction of a fluid with the surface of an object [5]. Wave drag is an additional resistance which occurs when an object moves through a liquid, e.g., during swimming or kayaking, due to the gravitational effects of the disturbance of the water-air interface [6]. Drag force is dominated by pressure drag which occurs when a body moves a quantity of fluid out of the way to pass through it [3]. This creates eddies that move downstream as a wake. If there is a faster flow with a less streamlined athlete, the flow separates further upstream and a larger wake with a lower pressure result in a larger difference in pressure between the front and the back of the athlete in the flow. The difference in pressure is the pressure drag which results in a net force which is acting opposite to the direction of the flow [6]. The wake behind the athlete results in less frontal pressure for the following athlete. This lower pressure zone provides a shelter from pressure drag by decreasing the differential acting across the athlete [3]. Furthermore, drag depends linearly on the density of the fluid and air density depends on air temperature, barometric pressure, and humidity. Changes in these variables have a proportionate effect on air density and, therefore, on the effect of drafting. Previous research has shown reductions of drag force for cycling from 38% [3] to 42% [7], speed skating of 23% [8], cross-country skiing of 25% [9], and running of 6.5% [10] and the passive drag for swimming was reduced by 13–26% [11]. Therefore, as the drag forces are reduced for the drafting athlete, a lower power output is required to overcome them. A considerable amount of research has been done on this topic related to successful performance in endurance sports. In professional sports, sometimes only 0.01 s makes the difference between winning and losing. Athletes can, therefore, gain advantage by perfecting their clothing, body posture (riding position), and equipment design and by using an optimal drafting strategy.

In the practical setting of sport, the biomechanical, physiological, and psychobiological parameters are of high interest because of their relation with sport performance. In terms of biomechanics, (external) power output (PO) is a common parameter [12]. Physiological parameters for monitoring physiological strain (i.e., the internal intensity) [13] have been indexed with the heart rate [14], lactate concentration [15], steady-state minute oxygen consumption (VO_2_) [16], minute ventilation (VE) [16], and electromyography (EMG) [17]. EMG measured the muscle activity, and the time slope of root mean square (RMS) is the outcome measure of the muscle activity. In addition, rating of perceived exertion (RPE), an often-reported psychobiological exertion parameter, is also used [18]. A linear relationship exists among the biomechanical, physiological, and psychobiological parameters and exercise intensity, meaning that a proportional reduction is observed in these parameters as exercise intensity is reduced [19]. Athletes can, therefore, take advantage of drafting as it can be used as an aid in pacing their performances.

Athletes can regulate their energy expenditure during a race by drafting to save energy for the final sprint [20]. Pacing is the goal-directed regulation of the exercise intensity over an exercise bout [21] and is the process of decision making for how and when to spend energy [22], resulting in a velocity distribution over a race [23]. Athletes can adjust their pacing strategy by more efficiently distributing their energy expenditure throughout a race. In addition, drafting can be used as a strategy to reduce energy expenditure during portions of the race, while maintaining velocity. Although drafting is not allowed in time trial races, in head-to-head competition or pack-style races, it is proven to be effective. Because of drafting, cyclists in a team or a group can maintain speeds significantly better than a single cyclist. Four riders alternating the leading position requires about 75% of the energy necessary for cyclists riding alone at the same speed [24]. In cycling, in team pursuit and pelotons, drafting has been found to benefit performance [25]. Similar benefits have been found in marathon running [26] and long-track speed-skating mass start events [27]. To date, there is limited research about how drafting can be applied as a part of a pacing strategy and in order to optimize performance.

Thus, because of reducing energy demands to maintain a certain velocity, drafting will have benefits for biomechanical, physiological, and psychobiological parameters, especially in the endurance sports, bicycling, kayaking, running, skating, cross-country skiing, and swimming. Through drafting, athletes may reduce their biomechanical, physiological, and psychological strains, as measured by PO, heart rate, lactate concentration, VO_2_, VE, EMG, and RPE. However, little is known about the magnitude of those parameters that can be achieved by drafting and the differences between sports. Insight into these benefits for the drafting athlete can help in improving the content for training, strategy for races, and ultimately improve performance. Even more, knowledge about the differences between the sports can help to improve our understanding of drafting as a pacing strategy in general and find out to what extend it is effective. Therefore, the purpose of this review is to identify the magnitude of drafting benefits for biomechanical, physiological, and psychobiological parameters in and between cycling, kayaking, running, skating, skiing, and swimming. It gives an overview of what is known about these parameters, which can lead to a better understanding on how athletes can benefit from drafting in order to increase their sport performances.

## 2. Methods

### 2.1. Literature Search

**Figure 1:**
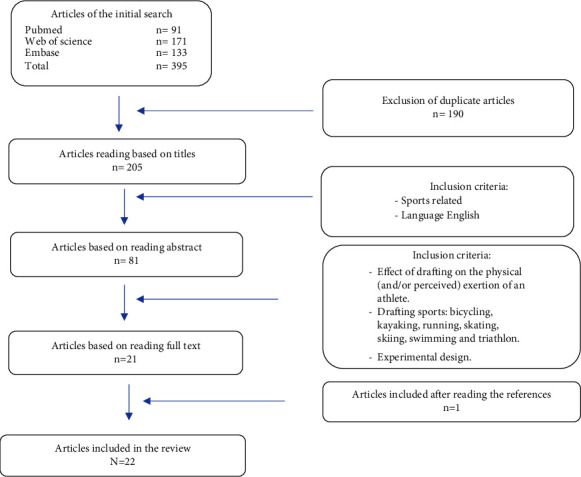
Flow diagram of the literature selection process and the number of articles (*n*) after each stage.

Systematic searches were performed in the following three databases: PubMed, Web of Science, and Embase for all literature until September 2022. Used search terms in these databases are shown in Table 1. The inclusion criteria for this systematic search were that studies should have investigated the effects of drafting on the biomechanical, physiological, and psychobiological parameters and that they were published in English. Exclusion criteria were that studies did not include an experimental design and that they did not include situations where the participants were in the leading as well as in the drafting position. The drafting sports bicycling, kayaking, running, skating, cross-country skiing, and swimming were included. Triathlon was also included in our search because it involves the sports cycling, running, and swimming which are included drafting sports in the current review. After the systematic search, 20 relevant articles were included as shown in Figure 1.

### 2.2. Outcome Measures

Biomechanical, physiological, and psychobiological parameters are taken into account in the studies and these parameters will be reviewed in this article. Biomechanical-included parameter is PO (watts). The physiological intensity-included parameters are the heart rate (beats per minute; BPM), blood lactate (mmol/L), VO_2_ (l/min or ml/kg/min), VE (l/min), and EMG (RMS). Last, a psychobiological parameter rating of perceived exertion (RPE; Borg scale: 6–20) was included.

### 2.3. Quality

All 22 included articles were evaluated for methodological quality using the critical review form quantitative studies from Law et al. [28], as shown in Table 2. This was done by two researchers and discussed until consensus was reached. The quality was assessed using 14 questions about study purpose, literature background, design of the study, sample size, methods, results, data analysis, practical importance, conclusions, and limitations of the study. Some questions were slightly adjusted for the purpose of this review. All questions used are formulated under Table 2. A score of 1 (meet the criteria) or 0 (does not meet the criteria) was given for each question. A score below seven points is considered of low methodological quality, between seven and ten of good quality and eleven and fourteen of high quality [48].

### 2.4. Statistical Analyses

First, an overview is made of all relevant articles. The meta-analyses were carried out for those articles, which contained information about the mean and SD of leading and drafting athletes. The analysis was carried out in Review Manager (RevMan 5.3, 2014). The standardized mean difference (SMD) [49] was calculated as a measure of effect for the differences between leading and drafting in each of the outcome measures using means and standard deviations (SDs). For articles that reported the standard error (SE) along with the mean, standard deviations were calculated (SD = SE ∗ √*n*). The effect sizes were weighted for the sample size. The *p* values were computed in a *Z*-test, which includes the effect estimate and the sample size. The cutoff for the significance of effect was set to *p* = 0.05 [50]. The benchmarks for effect sizes were set as trivial <0.2, small = 0.2–0.6, moderate = 0.6–1.2, large = 1.2–2.0, very large = 2.0–4.0, and extremely large >4.0 [50]. Heterogeneity was estimated with I^2^ in order to assess the extent (in percent), to which the included studies varied in their outcomes [50]. High heterogeneity implies a high variation among the studies in the comparison group/subgroup. The benchmarks for heterogeneity were set to low = 25%, moderate = 50%, and high = 75% [51]. Seven meta-analyses were performed, one for each outcome measure to test the overall effect of drafting. Furthermore, six meta-analyses were performed to test the moderating effect of sport, where it was hypothesized that the magnitude of the effect of drafting differs between sports. No moderator analysis was performed for PO and EMG as studies only reported on one sport for these parameters.

## 3. Results

### 3.1. Overview of the Articles

The search resulted in 205 studies whereof 22 studies were included following title and full-text screening.  Table 3 provides an overview of the included articles sorted by sport. The 22 articles are of high methodological quality and found strong evidence for the benefits of the drafting athlete. All included articles achieved a score of at least eleven points and as such are considered of high methodological quality. To conclude that there is strong evidence for the benefits of drafting, at least three high methodological quality studies with consistent results are needed [48]. Six studies involved road cycling [24, 38–40, 42, 44], of which two are from triathlon (triathlon sprint distance = 0.75 km swim, 20 km bike, and 5 km run) [38, 39] with a preceding swimming bout of 0.75 km. The remaining studies included one on kayaking [37], one on running [47], four on skating (two about inline skating [32, 43], one about short-track [46] speed skating, and one in long-track speed skating) [32], two on cross-country skiing (using different techniques) [39, 40], and eight on swimming [11, 29, 33–36, 45], of which two are from triathlon research [35, 36]. One study included 28 participants [42], and the remaining studies included 18 participants or less. In total, 232 participants were included which resulted in 548 observations in total.

Two types of nondrafting were used in the studies, namely, drafting versus leading or drafting versus alone. Leading means that the athlete is in front but is followed by one or more other athletes behind. In seventeen studies, significant reductions were found for the drafting situation, and five studies found no difference between leading and drafting in one parameter or more [11, 34, 39, 44, 47].

### 3.2. Meta-Analysis

**Figure 2:**
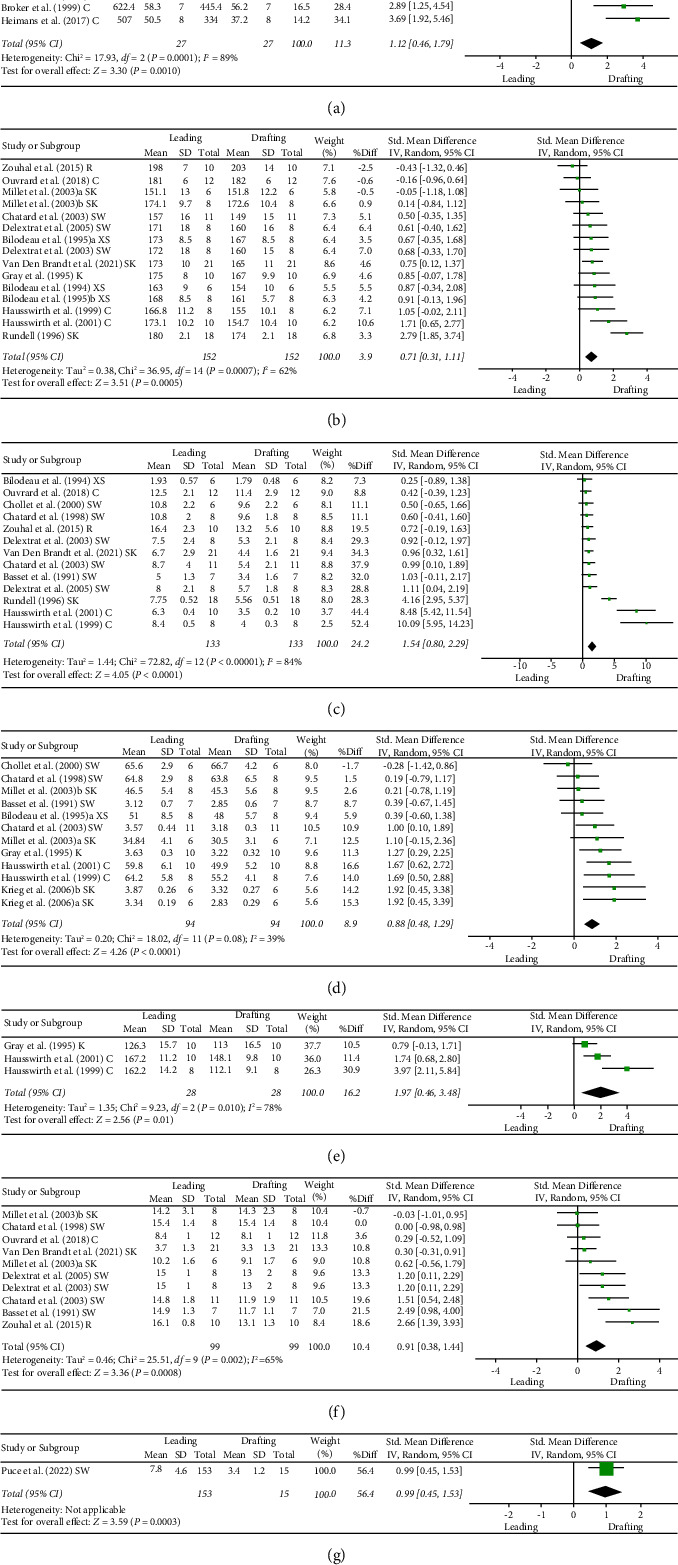
Comparison between leading and drafting situations of the included studies in (a) PO (Watt), (b) heart rate (beats per minute), (c) blood lactate (Mmol/L), (d) VO_2_ (ml/min/kg or l/min), (e) VE (ml/min/kg), (f) RPE, and (g) EMG. C=cycling; K = kayaking; R = running; SK = skating; XS = cross-country skiing; SW = swimming.

In total, 21 of the 22 articles included information about the mean and SD and were included in the meta-analysis.  Figure 2 as well as Table 4 show the results of the meta-analyses in forest plots, including PO, heart rate, blood lactate, VO_2_, VE, RPE, and EMG. The meta-analyses for all biomechanical, physiological, and psychobiological parameters indicate that drafting results in lower intensities compared to leading. The weighted differences are for PO (11.3%), heart rate (3.9%), blood lactate (24.2%), VO_2_ (8.9%), VE (16.2%), RPE (10.4%), and EMG (56.4%). Moderate effect sizes and moderate heterogeneity were found for the heart rate (ES = 0.71; CI 95% = 0.31–1.11; and *p* ≤ 0.001), VO_2_ (ES = 0.88; CI 95% = 0.48–1.29; and *p* < 0.001), and EMG (ES = 0.99; CI 95% = 0.45–1.53; and *p* < 0.001). Large effect sizes and high heterogeneity were found for blood lactate (ES = 1.54; CI 95% = 0.80–2.29; and *p* ≤ 0.001) and VE (ES = 1.97; CI 95% = 0.46–3.48; and *p* = 0.01). Moderate effect sizes and high heterogeneity were found for PO (ES = 1.12; CI 95% = 0.46–1.79; and *p* = 0.001) and RPE (ES = 0.91; CI 95% = 0.38–1.44; and *p* ≤ 0.001).

The results of the moderator analysis for the variable sport are presented in Table 4. Significant heterogeneity was found for the heart rate (*I*^2^ = 62% and *p* < 0.001), blood lactate (*I*^2^ = 84% and *p* < 0.001), ventilation (*I*^2^ = 78% and *p* = 0.01), and RPE (*I*^2^ = 67% and *p* = 0.002). Differences in the effect in studies within these parameters can be explained by sport. No significant differences were found for the remaining parameters.

## 4. Discussion

The purpose of this review was to identify the magnitude of drafting benefits for biomechanical, physiological, and psychobiological parameters in and between cycling, kayaking, running, skating, skiing, and swimming. The results of the meta-analyses show that drafting has a significant effect on the physiological strain. The effect of drafting on the included parameters was found to be moderately large overall. Although the magnitude of the effect varies among the intensity parameters and sports, there is strong evidence that drafting reduces the biomechanical, physiological, and psychobiological effects on the athlete. This reduction for the drafting athlete is a general positive effect across all outcome measures. Athletes in cycling seem to benefit the most whereas the research on athletes in running shows an inconsistency with a negative effect for the heart rate and positive effects for blood lactate and RPE. The intensity parameter ventilation resulted in the highest effect size.

In head-to-head competitions, opponents compete directly against each other for the win. When drafting is allowed, it is used by the athletes to reduce air resistance by positioning themselves behind teammates or opponents. During team pursuit races in cycling, for example, where team members together need to be faster than their opponents, drafting is crucial for the performance of the team [24]. When competing directly against others, athletes adjust their pacing strategy to that of their opponents [21]. This implies that the competing athletes influence each other's pacing strategy by choosing the optimal tactical position for an energetically optimal profile [20]. It was suggested by earlier research during speed-skating mass start [27] that drafting during the race is a strategic and tactical social dilemma because all skaters want to hide behind others to save energy. As such, athletes regulate their exercise intensity differently in head-to-head competition compared to time trial races [21]. Research in swimming even called drafting an effective tactic [45]. The adaptation of pacing behaviour towards external factors (such as other athletes) has been shown to provide a greater physiological challenge compared to self-paced exercise [52]. The reason is that the possibility of drafting draws the athlete away from the energetically favorable strategies as would be performed in time-trial exercise. Drafting is, therefore, an interesting and complex but important element of pacing behaviour and tactical decision making [21]. Still, this has only marginally been taken into account within the literature of pacing even though it is an important aspect. It is important to realize that behavioural adaptations in pacing differ between sports, for example, between cycling and speed skating. As such, optimal pacing needs to be studied sport specific [53]. In short-track speed skating and cycling, which have a high beneficial effect of drafting, the race was characterized by relatively slow development. But in running, in which a relatively low beneficial effect of drafting was found, the athletes tend to adopt a pacing strategy in the beginning of the race that they cannot sustain until the end [21]. It seems important to look carefully at the unique characteristics of the different sports and disciplines before making the optimal drafting plan within the pacing strategy.

As expected, differences were found in the effects of drafting among the included sports. This is due not only to the tactical (i.e., pacing) aspects but also to the different technical and biomechanical aspects of the different sports. The magnitude of the effects among the sports can be found in the moderator analysis which is presented in Table 4. All sports except running are consistently benefitting from drafting. Cycling seems to benefit the most. Running shows an inconsistency with a negative effect for the heart rate and positive effects for blood lactate and RPE.

Drafting is particularly effective for reducing the physiological strain in cycling with large effect sizes for PO, blood lactate, VO_2_, and VE. In cycling, it is relatively easy to maintain a close position behind the leader and cycling is conducted at high velocities. As cyclists can monitor their own rhythm and PO on the pedals, they are able to maintain an even velocity with the leader [40]. However, certain skills and courage are required to draft as close as possible within the wheels of the peloton [25]. In speed skating, the athletes also move in an aerodynamic posture but as different postures have different frontal areas, skaters need to adapt their posture, stroke frequency, and skating technique to maintain a similar rhythm as the leader in order to gain the full benefits of drafting [54]. Furthermore, as speed skating is performed on a track, skaters frequently round corners that require them to cross their legs, which make it difficult to maintain a close distance to the leader as the rhythm is frequently altered and there is an increased risk of falling [46]. This factor is amplified in short-track speed skating, which is performed on a 111 m track with more frequent corners of a smaller radius as opposed to speed skating, which is performed on a 400 m track. Cross-country skiers also need to adapt the rhythm of their skiing technique to each other while drafting [31]. Cross-country skiers spend less time than speed skaters in a crouched position and more time in an upright standing position. Therefore, the frontal area or the anthropometrics of the athletes seem even more important when, for example, forming a team for competition or making training partners in order to gain most benefits from drafting. It has also been found that swimmers need to adapt their rhythm to the leader and to maintain a close distance to the leader when propelling themselves with a fully immersed body [11]. The immersed body while swimming makes it challenging to compare to the other sports because swimmers experience drag in water instead of air environment. The drafting athlete moves in the wake of the leading athlete and can take advantage of the water turbulence of the leader [7]. This also counts for kayaking. Swimming, though, is performed at lower speeds than the other sports. Only running found an inconsistency regarding the negative effect for heart rate. As in swimming, this sport is performed at a lower velocity and a potential explanation for the adverse effect of drafting regarding the heart may be found in thermoregulation. When drafting closely behind another athlete, it may be more difficult to release heat resulting in a higher heart rate frequency. However, more research is needed. While running, which is performed in an upright position, athletes have more difficulty to stay closely behind the leader and this might cost extra energy [47]. In addition, when comparing the different sports, the surface material (e.g., different types of ice, snow, and asphalt), which differs between the included sports, and the type of materials (e.g. skates, skies, tires, and boats) used all have an impact on the magnitude of the drafting effect.

Knowing that drafting not only gains benefits but also knowing the magnitude of the benefits can be influenced by the athlete's ability to “draft properly” is important knowledge to improve training together and also for improving race strategies and eventually race performances. To “draft properly” is a wide concept. The included articles in this review discussed multiple important aspects that can influence the drafting effect. The velocity of the athletes, distance between the athletes, body size, position, and number of riders in the team and drafting skills of the athletes are all found to be relevant while drafting. Most of the aspects are difficult to investigate in practical settings of sports and not all aspects are included in every single article or in all different sports. To start with the velocity, wind tunnel research at drag reductions concluded that the drafting effect is magnified with greater velocity [3] but research to internal and external intensity in the practical setting is inconclusive on this topic. A cycling study compared different velocities and concluded that the benefits of drafting in terms of VO_2_ reductions were greater when cycling at 37 or 40 km/h (28% and 26%) than at 32 km/h (18%) [42]. But inline speed skating studies found different results; the effect of drafting on VO_2_ at 30 km/h (15.3%) was similar to 33 km/h (14.2%) [41]. Even the opposite was found, namely, lower reductions for VO_2_ for higher velocity of 25.2 km/h (2.7%) comparing to higher reductions at a lower velocity of 19.8 km/h (9.6%) [43]. Cycling and inline speed skating are different sports and the inline speed skating studies included participants from the recreational level and used lower velocities that in the cycling study, which may explain the results. But more research into this topic is needed to form clearer conclusions about the effect of different velocities on the internal and external intensity of athletes in different sports. This is also the case with the distances between the athletes. It is important to draft as close as possible behind the leader because the drag is then the lowest as found in wind tunnel research for cyclists and skaters [3, 40, 54]. But this was not found in the inline skating study [43], in which participants skated as close as possible to the leader and on a distance of “an outstretched arm” from the leader. For the body size, wind tunnel research in cycling showed more benefits when drafting behind a bigger leader compared to drafting behind a smaller leader, relating to the previously mentioned importance of the frontal area [1]. A cycling study found similar results and concluded that when composing a team, it is preferred to select athletes with similar anthropometrics because the frontal area was found to have an impact on the effect of drafting [40]. If this is also the case in other sports is still unknown due to the lack of research to the frontal area in the practical settings. With regards to the rider position in the team in cycling studies [26, 40] and the runner position in the group [48], it has been established that the bigger the team, the bigger the advantage. Finally, a very important aspect of drafting is that it is a skill. Drafting as a skill is difficult to measure but is seen as an important aspect as the ability to apply it has a significant impact on the extent to which the effects of drafting discussed in the current article impact performance. Drafting skills may be more important than how fast skaters can skate on individual races and it may be a key factor in order to win the race [27]. To highlight the importance of drafting, it is unlikely for cyclists who are not able to take part in a peloton to win. This is also true for swimmers who cannot maintain close distance to the leader or cross-country skiers who have difficulties in adapting the technique of the leading skier.

In training settings, the goal of the session decides whether drafting should be used or not to optimize the training adaptations. For example, drafting allows for training at higher velocities and for more repetitions whilst maintaining a reduced or equal physiological strain. Nondrafting is beneficial for training at a higher intensity. The only article that included EMG measurements was in swimming and stated that a drafter can profit from a leader of equal and of a superior level. Drafting an equal level leader allows better energy management and a superior leader elevates their pace beyond their normal ability [45]. From all the included physio(bio)logical strain parameters, heart rate shows the lowest reduction for drafting and blood lactate the highest. Therefore, it may be useful to not only rely on one parameter but also if possible, include measures for other physiological parameters simultaneously (e.g., measuring both heart rate and PO simultaneously during a training while cycling). Youth athletes learn better, so implementing drafting in training at a young age will improve performances even more.

This review focused on the research about the effect of drafting on the physio(bio)logical strain of athletes in the practical setting of sports. In this way, conclusions can directly relate to sport settings, in training or competition environments, in order to improve performances for athletes. Because of the exclusion criteria, quite a large number of articles that used computational fluid dimensional simulations or lab studies were excluded. The included literature focused on fieldwork to characterize impact in the sport environment, but the 22 included articles all point in the same direction; drafting results in benefits for the drafting athletes in the field. Because of the reduction in drag for the drafting athlete, this was expected, but the magnitude of the effect in terms of biomechanical, physiological, and psychobiological intensities which results from this review is relevant information for athletes and gives more understanding into the importance of the benefits of drafting. This review shows that in literature, the focus is mainly on physiological parameters which shows a gap in the literature on especially the psycho(bio)logical strain. Furthermore, methodological quality was high for all the included articles which results in a very strong evidence. This review is the first to sum up the benefits of drafting in different sports. All sports except running are consistently benefitting from drafting. Cycling seems to benefit the most. Running shows an inconsistency with a negative effect for heart rate and positive effects for blood lactate and RPE.

### 4.1. Limitations and Future Research

Because this review focused on research that was done in the practical setting, a limited number of articles was included. However, this made the current review ecologically valid. For some sports, only a limited number of studies could be included. For example, there was only one study about running and one about kayaking. More studies were recruited for the remaining sports. Therefore, the results may not be comparable to the same extent in the different sports. Also, the kayaking study [37] stated that practice and implementation of drafting situations under velocities similar to the competition velocity can reduce energy expenditure. Therefore, the magnitude of the drafting benefits could have been even higher when the study participants would have been on the competitive performance level. Also, the lack of drafting skills and the participation of recreational athletes in some included articles may have lowered the demonstrated benefits of drafting. To further unravel the benefits of drafting, more research with highly competitive athletes would be highly recommended.

Lastly, rating of perceived exertion points in the same direction as all the other internal intensity parameters, but literature on this psychobiological parameter is most scarce. Therefore, more research into drafting is necessary in the practical setting of sports, focusing also on psychobiological intensity parameters and executing more in the competition environment, using elite athletes and competition velocities to improve the research of drafting in practice. This review made the first step in unraveling the drafting puzzle but future research is necessary in order to make the next valuable steps in improving drafting in practice.

### 4.2. Perspective

For everyone involved in sports where drafting is permitted or can be used, it is of high importance to be aware of the significant benefits of drafting. It reduces physiological stress and strain of drafting athletes and the magnitude of the drafting effect should not be underestimated as the difference of the magnitude between sports. The benefits of drafting can improve training sessions by using training partners for drafting or nondrafting situations to use drafting as a tactic in head-to-head competition in order to save energy for the final sprint and to improve performance of a team by composing the most efficient team on anthropometrics and drafting skills. Despite that, more research is needed into drafting in the practical setting or competition environment as there are drafting skills that have influence on the magnitude of the drafting effect. The factors to keep in mind when optimizing drafting strategies are the velocity of the athletes, distance between the athletes, body size, position and number of riders in the team, and drafting skills of the athletes. These are all found to be relevant while drafting. Therefore, paying attention to these factors while drafting during training can help improve athletes' pacing strategies and their performance.

## 5. Conclusion

In conclusion, findings from 22 articles within seven sports involving 214 participants showed a significant reduction for physio(bio)logical strain for the drafting athlete in all sports. The moderator analysis showed differences between sports on the magnitude of the effect of drafting, so the essence of the sport is important to take into account. Although the magnitude of the effect varies among intensity parameters and sports, the results suggest that drafting can reduce the biomechanical, physiological, and psychobiological effects on the athlete. Drafting can be considered as an aspect that can be influenced to optimize pacing strategy to save energy. Therefore, it can improve training sessions, race strategies, and even performance in competition. It is advised to be aware that drafting skills also influence the magnitude of the drafting effect and to use this knowledge to take advantage of drafting.

## Figures and Tables

**Table 1:** Search terms used in the databases PubMed, Web of Science, and Embase.

PubMed	Web of Science	Embase
(“Sports”[Mesh:NoExp] OR “Bicycling”[Mesh] OR “Skiing”[Mesh] OR “Water Sports”[Mesh] OR “Running”[Mesh] OR bicycl∗[tiab] OR cycli∗[tiab] OR skiing[tiab] OR skating[tiab] OR running[tiab] OR swimming[tiab] OR kayak∗[tiab] OR triathlon[tiab]) AND (drafting[tiab] OR slipstream∗[tiab] OR slip-stream∗[tiab] OR al position[tiab])	TS = (“bicycl∗” OR “cycli∗” OR “skiing” OR “skating” OR “running” OR “swimming” OR “triathlon” OR “kayaking”) AND TS = (“drafting” OR “slipstream∗” OR “slip-stream∗” OR “sheltered position”)	(“sport”/de OR “cycling”/exp OR “jogging”/exp OR “roller skating”/exp OR “running”/exp OR “skating”/exp OR “skiing”/exp OR “swimming”/exp OR “triathlon”/exp OR “rowing”/exp OR (bicycl∗ OR cycli∗ OR skiing OR skating OR running OR swimming OR triathlon OR kayak∗):ab,ti) AND (drafting OR slipstream∗ OR “slip-stream∗” OR “sheltered position”):ab,ti

**Table 2:** Methodological quality of the reviewed articles.

Authors, years	Question														
	1	2	3	4	5	6	7	8	9	10	11	12	13	14	Total
Basset et al., 1991 [29]	1	1	1	1	1	1	1	1	1	1	1	0	1	0	12
Bilodeau et al., 1995 [30]	1	1	1	1	1	1	1	1	1	1	1	0	1	0	12
Bilodeau et al., 1995 [31]	1	1	1	1	1	1	1	1	1	1	1	0	1	0	12
Van den Brandt et al., 2021 [32]	1	1	1	1	1	1	1	1	1	1	1	1	1	1	14
Broker et al., 1999 [24]	1	1	1	1	1	1	1	1	0	1	1	0	1	0	11
Chatard et al., 1998 [11]	1	1	1	1	1	1	1	1	1	1	1	0	1	0	12
Chatard et al., 2003 [33]	1	1	1	1	1	1	1	1	1	1	1	0	1	0	12
Chollet et al., 2000 [34]	1	1	1	1	1	1	1	1	1	1	1	0	1	0	12
Delextrat et al., 2003 [35]	1	1	1	1	1	1	1	1	1	1	1	0	1	0	12
Delextrat et al., 2005 [36]	1	1	1	1	1	1	1	1	1	1	1	0	1	0	12
Gray et al., 1995 [37]	1	1	1	1	1	1	1	1	1	1	1	0	1	1	13
Hausswirth et al., 1999 [38]	1	1	1	1	1	1	1	1	1	1	1	0	1	0	12
Hausswirth et al., 2001 [39]	1	1	1	1	1	1	1	1	1	1	1	0	1	0	12
Heimans et al., 2017 [40]	1	1	1	1	1	1	1	1	1	1	1	0	1	1	13
Janssen et al., 2009 [6]	1	1	1	1	1	1	1	1	1	1	1	0	1	0	12
Krieg et al., 2006 [41]	1	1	1	1	1	1	1	1	1	1	1	0	1	1	13
McCole et al., 1990 [42]	1	1	1	1	1	1	1	1	1	1	1	0	1	0	13
Millet et al., 2003 [43]	1	1	1	1	1	1	1	1	1	1	1	0	1	0	12
Ourvrard et al., 2018 [44]	1	1	1	1	1	1	1	1	1	1	1	0	1	1	13
Puce et al., 2022 [45]	1	1	1	1	1	1	1	1	1	1	1	1	1	1	14
Rundell., 1996 [46]	1	1	1	1	1	1	1	1	1	1	1	0	1	0	12
Zouhal et al., 2015 [47]	1	1	1	1	1	1	1	1	1	1	1	0	1	0	12

First authors are mentioned; 1 = meet the criteria or 0 = does not meet the criteria. (1) Was the aim of the study stated clearly? (2) Was relevant background literature reviewed? (3) Was the design appropriate for the research question? (4) Was the sample described in detail? (5) Was informed consent obtained? (6) Were the outcome measures reliable and valid? (7) Was the intervention described in detail? (8) Was a contamination and cointervention avoided? (9) Were results reported in terms of statistical significance? (10) Were the analysis methods appropriate for the research design? (11) Was practical importance reported? (12) Were dropouts reported? (13) Were conclusions appropriate given the study findings? (14) Were limitations of the study acknowledged and described by the authors?

**Table 3:** Overview of articles on drafting with author names, number of participants with age in years, level of performance of the participants, drafting versus leading or drafting versus alone, design of the study, parameters, velocity, and practical implications of all the included articles.

Authors, years	Number of participants and gender	Age in years	Level of performance	Nondrafting situation	Design	Parameters	Velocity (km/h)	Practical implications
*(Bi)cycling*								
Broker et al., 1999 [24]	7 males	NR	International	Leading	2 km	Power output	60 km/h	
Hausswirth et al., 1999 [38]	8 males triathletes	20.8 ± 2.1	International	Alone	2 × 20 km	Heart rate, blood lactate, VO_2_, VE	39.5 km/h	
Hausswirth et al., 2001 [39]	10 males triathletes	25.6 ± 4.1	NR	Leading	2 × 20 km	Heart rate, blood lactate, VO_2_, VE	41 km/h	
Heimans et al., 2017 [40]	8 males	23.9 ± 3.4	International	Leading	10 × 3 km	Power output	56.7 km/h	Selection of most potential cyclists for the team
McCole et al., 1990 [42]	28 males	NR	NR	Alone	2 km	VO_2_	32 km/h 37 km/h 40 km/h	
Ouvrard et al., 2018 [44]	12 males	23 ± 3.7	Recreational	Alone	2 × 2.7 km	Heart rate, blood lactate, power output, and RPE	Leader 18.8, drafter 18.0 km/h, significant difference 4.2%	Leading teammate improves uphill cycling performance

*Kayaking*								
Gray et al., 1995 [37]	10 males	25 ± 6.5	International	Alone	2x km	Heart rate, VO_2_, VE	13.32 km/h	Practice and implementation drafting reduced energy expenditure on competition speeds

*Running*								
Zouhal et al., 2015 [47]	10 males	25.6 ± 3.1	National	Alone	2 × 3 km	Heart rate, blood lactate, power output	Velocity significant different	Time benefit, design of training program, competitive strategies, and psychological benefits

*Skating*								
Van den Brandt et al., 2021 [32]	12 males and 10 females	19.3 ± 2.6	International/national	Leading and alone	4 × 1.2 km	Heart rate, blood lactate, RPE	37.2–39.5 km/h	RPE equal for drafting and leading compared to skating alone
Krieg et al., 2006 [41]	6 males	25 ± 6	National	Leading	2 × 6 min	VO_2_		
A							30 km/h	
b							33 km/h	
Millet et al., 2003 [43]	8 males	33.3 ± 7.6	Recreational	Alone	6 × 6 min	Heart rate, VO_2_, RPE		
a							19.8 km/h	
b							25.2 km/h	
Rundell, 1996 [46]	14 males and 4 females	20.3 ± 4.1	National	Leading	2 × 4min	Heart rate, blood lactate	33.1 km/h	Drafting technique should be trained

*Cross-country skiing*								
Bilodeau et al., 1994 [39]	6 males	24.8 ± 5.6	NR	Leading	2 × 2 km	Heart rate, blood lactate	20.2 km/h	Following a skier bigger than yourself better than a smaller skier
Bilodeau et al., 1995 [40]	8 males and 2 females	27 ± 2	NR	Leading	2 × 2 km technique: DS DP DS DP	Heart rate, VO_2_	Males 17.7 km/h Males 17.7 km/h Females 16 km/h Females 16 km/h	Following a skier bigger than yourself better than a smaller skier. And drafting is more advantageous in the presence of head wind

*Swimming*								
Basset et al., 1991 [29]	7 males	36 ± 3	NR	Alone	2 × 0.549 km	Heart rate, blood lactate, VO_2_, RPE	4.3 km/h	In competitive swim training drafting should be avoided to maximize swim training benefits
Chatard et al., 1998 [11]	8 males	24.4 ± 2.7	International/national	Alone	2 × 0.4 km	Blood lactate, VO_2_, RPE	4.8 km/h, 5 km/h, significant different 3.2%	Train on the drafting technique and the racing strategy
Chatard et al., 2003 [33]	11 males	24 ± 5	NR	Alone	5 × 4min	Heart rate, blood lactate, VO_2_, RPE	4.3 km/h	
Chollet et al., 2000 [34]	6 males	24.7 ± 1.3	International	Alone	0.4 km	Blood lactate, VO_2_,	4.8 km/h, 5 km/h, significant different	Drafting contributes to stabilizing the stroke parameters
Delextrat et al., 2003 [35]	8 males	26 ± 6	Interregional/national	Alone	2 × 0.75 km	Heart rate, blood lactate, RPE	4.2 km/h	
Delextrat et al., 2005 [36]	8 males	27 ± 6	Interregional/national	Alone	2 × 0.75 km	Heart rate, blood lactate, RPE	4.1 km/h	
Janssen et al., 2009 [6]	4 males, 5 females	29.4 ± 11.2	NR	Leading	2 × 4 min	VO_2_	4.3 km/h	Swim as close as possible to the leader
Puce et al., 2022 [45]	14 males, 4 females	20.1 ± 3.1	Interregional/national	Leading	3 × 0.2 km	EMG	NR	Drafter can profit from an equal level or superior level leader

^#^Use alone instead of leading. NR = not registered. Age in years (mean ± SD). Velocity in kilometer per hour.

**Table 4:** The variation between sports in the effect of drafting on the outcome measures: heart rate, blood lactate, oxygen uptake (VO_2_), ventilation (VE), rating of perceived exertion (RPE), and electromyography (EMG).

	*n* studies	*n* participants	% diff.	ES (CI 95%: upper, lower)	*p* (*Z*-testoverall effect)	Heterogeneity *I*^2^(*p*)
**Heart rate**	**15**	**152**	**3.84**	**0.71 [0.31, 1.11]**	**0.005**	**62% (<0.001)**
Skating	4	53	3.09	0.92 [−0.26, 2.10]	0.13	85% (<0.001)
Kayaking	1	10	4.57	0.85 [−0.07, 1.78]	0.34	^*∗*^
Cycling	3	30	5.09	0.82 [−0.32, 1.96]	0.16	76% (0.02)
Cross-country skiing	3	22	4.26	0.81 [0.19, 1.43]	0.01	0% (0.94)
Swimming	3	27	6.08	0.58 [0.04, 1.13]	0.04	0% (0.96)
Running	1	10	−2.53	−0.43 [−1.32, 0.46]	0.34	^*∗*^
**Blood lactate**	**13**	**133**	**26.03**	**1.54 [0.80, 2.29]**	**<0.001**	**84% (<0.001)**
Cycling	3	30	27.26	6.14 [−0.76, 13.04]	<0.001	95% (<0.001)
Skating	2	39	31.31	2.52 [−0.61, 5.65]	0.11	95% (<0.001)
Swimming	6	48	24.71	0.87 [0.44, 1.29]	<0.001	0% (0.96)
Running	1	10	19.51	0.72 [−0.19, 1.63]	0.12	^*∗*^
Cross-country skiing	1	6	7.25	0.25 [−0.89, 1.38]	0.67	^*∗*^
**VO**_**2**_	**12**	**94**	**9.11**	**0.88 [0.48, 1.29]**	**<0.001**	**39% (0.08)**
Cycling	2	18	15.38	1.68 [0.89, 2.47]	<0.001	0% (0.98)
Kayaking	1	10	11.29	1.27 [0.29, 2.25]	0.01	^*∗*^
Skating	4	26	10.09	1.16 [0.30, 2.02]	0.008	46% (0.14)
Swimming	4	32	5.76	0.39 [−0.13, 0.92]	0.15	8% (0.35)
Cross-country skiing	1	8	5.88	0.39 [−0.60, 1.38]	0.44	^*∗*^
**Ventilation**	**3**	**28**	**17.12**	**1.97 [0.46, 3.48]**	**0.01**	**78% (0.01)**
Cycling	2	18	19.93	2.72 [0.55, 4.89]	0.01	76% (0.04)
Kayaking	1	10	10.53	0.79 [−0.13, 1.71]	0.09	^*∗*^
**RPE**	**9**	**87**	**11.72**	**1.00 [0.41, 1.60]**	**<0.001**	**67% (0.002)**
Running	1	10	18.63	2.66 [1.39, 3.93]	<0.001	
Swimming	5	42	13.69	1.19 [0.45, 1.92]	0.002	55% (0.07)
Skating	3	35	7.06	0.27 [−0.20, 0.75]	0.26	0% (0.70)

^*∗*^Heterogeneity not estimable, only one study in group; ES = effect size; CI = confidence interval; *I*^2^ = heterogeneity. The outcome measures are displayed in bold.

## Data Availability

No data were used to support this study.
